# Biweekly cetuximab in combination with capecitabine and oxaliplatin (XELOX) or irinotecan (XELIRI) in the first-line and second-line treatment of patients with RAS wild-type metastatic colorectal cancer

**DOI:** 10.3332/ecancer.2022.1490

**Published:** 2022-12-15

**Authors:** Jamal Zekri, Mohammed Abbas Baghdadi, Refaei Belal Ibrahim, Abdelrazak Meliti, Turki M Sobahy

**Affiliations:** 1 College of Medicine, Al-Faisal University, Riyadh 11533, Saudi Arabia; 2 King Faisal Specialist Hospital & Research Centre (Jeddah), Jeddah 21499, Saudi Arabia; 3 Research Centre, King Faisal Specialist Hospital & Research Centre (Jeddah), Jeddah 21499, Saudi Arabia; 4 Department of Clinical Oncology and Nuclear Medicine, Faculty of Medicine, Al Azhar University, Cairo 11884, Egypt; 5 Department of Pathology and Laboratory Medicine, King Faisal Specialist Hospital & Research Centre, Jeddah 21499, Saudi Arabia

**Keywords:** biweekly cetuximab, XELOX, XELIRI, colorectal cancer, anti-EGFR

## Abstract

**Background:**

Oral capecitabine in combination with intravenous oxaliplatin (XELOX) or irinotecan (XELIRI) are acceptable substitutions to fully intravenous regimens. Biweekly (as opposed to weekly) cetuximab is more convenient when combined with biweekly chemotherapy. Here, we report the tolerability and efficacy of biweekly cetuximab in combination with biweekly XELOX or XELIRI in patients with RAS wild-type metastatic colorectal cancer (RAS-WT mCRC).

**Methods:**

Clinical data of consecutive patients with mCRC who received biweekly cetuximab (500 mg/m^2^) in combination with XELOX or XELIRI between January 2009 and May 2019 in the first- or second-line settings was extracted. Dosage of XEL (Capecitabine/XELODA) was 1,000 mg/m^2^ twice daily for 9 days, plus on day 1 oxaliplatin 85 mg/m^2^ or irinotecan 180 mg/m^2^. Treatment dose reduction and delay for ≥7 days was analysed as surrogates for toxicity. Extended RAS testing was performed in the context of this study for patients who received treatment based on limited KRAS-WT genotype.

**Results:**

Sixty one patients with RAS-WT mCRC fulfilled the eligibility criteria. XELOX was administered to 26 (42.6%) and XELIRI to 35 (57.4%) of patients. For all patients in the first-line setting, the objective response rate (ORR), median progression free survival (PFS) and median overall survival (OS) were 54%, 8 months and 25 months, respectively. The corresponding outcomes for the subgroup of patients who received first-line XELOX were 68%, 10 months and not reached, respectively. For all patients in the second-line setting, the ORR, PFS and OS were 50%, 7 months and 20 months, respectively. Chemotherapy components dose reduction and delays were observed in 18 (29.5%) and 25 (41%) patients, respectively. The corresponding frequencies for cetuximab were 3 (5%) and 31 (50.8%).

**Conclusion:**

Biweekly cetuximab in combination with XELOX or XELIRI is tolerable and effective. The addition of cetuximab to capecitabine and oxaliplatin is associated with favourable outcome.

## Introduction

Recent reports based on Global Cancer Incidence, Mortality And Prevalence (GLOBOCAN) 2020 data estimate 19.3 million new cancer cases globally with colorectal cancer (CRC) representing 10% of these cases preceded only by lung (11.4%) and breast (11.7%) cancers [1].

Some countries are reporting stability or slight decline in the incidence of CRC, possibly due to screening. However, others are experiencing a significant rise in reported cases [2, 3].

Approximately a quarter to third of patients with CRC present with metastatic disease and up to 50% develop metastases after initial curative intent treatment [4, 5].

The outcome for patients with metastatic CRC (mCRC) is poor. However, there has been a measurable improvement in outcome over the recent two decades due to the adoption of the multidisciplinary team management and the successful development of newer and more effective chemotherapy and targeted agents [6, 7].

Infusional 5 Fluorouracil (5FU) is the most commonly used fluoropyrimidine backbone cytotoxic agent in landmark trials and in daily routine practice. The more convenient oral fluoropyrimidine, capecitabine is an attractive alternative to infusional 5FU as monotherapy and in combination with oxaliplatin and irinotecan in 3-weekly and biweekly regimens [8, 9].

Cetuximab is an IgG-1 monoclonal antibody that binds to and inhibits the epidermal growth factor receptor (EGFR). Randomised phase III trials investigated the addition of cetuximab to 5FU-based combination chemotherapy regimens (FOLFOX/FOLFIRI) in mCRC. Results of these studies confirmed the clinical activity of this approach in patients with RAS wild type (RAS-WT) tumours [10–12].

Currently, management guidelines endorse the use of anti-EGFR antibodies such as cetuximab in combination with chemotherapy in patients with RAS-WT mCRC in the first and subsequent lines of treatment.

The approved schedule of cetuximab consists of first a loading dose of 400 mg/m^2^ followed by 250 mg/m^2^ every week. Meanwhile, most chemotherapy regimens for the treatment of mCRC are administered biweekly. This creates frequent visits to the hospital and extra costs. Pharmacokinetic, pharmacogenomic and pharmacoproteomic studies show equivalence of weekly (250 mg/m^2^) and biweekly (500 mg/m^2^) administration [13].

Substituting weekly with biweekly cetuximab in combination with biweekly capecitabine based combination chemotherapy provides the prospect of convenience and cost effectiveness both for the patient and health care providers.

Encouraged by the above, we adopted biweekly regimens of cetuximab in combination with capecitabine and oxaliplatin (XELOX) and with irinotecan (XELIRI) in routine clinical practice. Here we report our experience with these regimens coupled with planned and structured evaluation of efficacy and tolerability.

## Patients and methods

This was a single centre, non-randomised retrospective study. All consecutive patients with mCRC who received biweekly cetuximab (500 mg/m^2^) in combination with chemotherapy for mCRC were identified through the pharmacy records. Additional inclusion criteria were as follows: (a) the chemotherapy component of the regimen contains capecitabine combined with either oxaliplatin or irinotecan administered every 2 weeks and (b) treatment in the first- or second-line settings. Exclusion criteria were as follows: (a) chemotherapy component of the regimen containing infusional 5FU, (b) chemotherapy component regimen lacking fluoropyrimidine such as single-agent iriniotecan or cetuximab, (c) treatment in third or subsequent line settings and (d) receiving cetuximab in both first- and second-line settings. The above inclusion and exclusion criteria were designed to specifically select patients who received biweekly cetuximab combined with biweekly capecitabine and oxaliplatin (XELOX) or capecitabine and irinotecan (XELIRI). Dosage of capecitabine was 1,000 mg/m^2^ twice daily for 9 days, oxaliplatin 85 mg/m^2^ on day 1 and irinotecan 180 mg/m^2^ on day 1 administered every 2 weeks.

The sample size is not based on statistical considerations and it represents the number of eligible patients who started treatment with the regimens of interest between January 2009 and May 2019. The study was approved by the Institutional Review Board.

**Figure 1.**
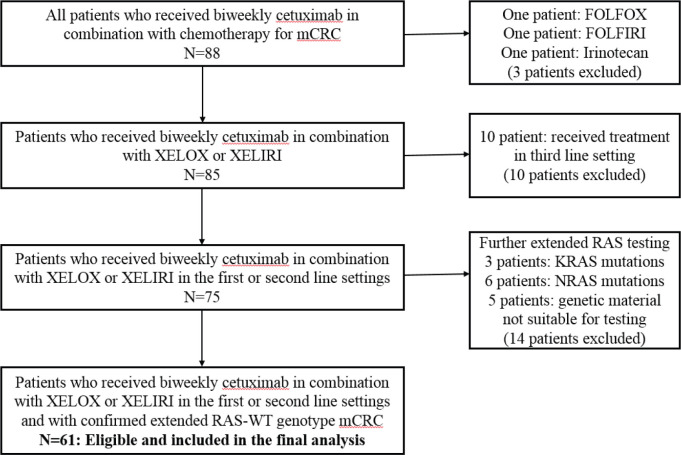
Study flow diagram.

Due to the historic nature of the study, some patients received cetuximab based on limited RAS results (only exon 2 KRAS-WT was confirmed). For the purpose of this study, additional KRAS and NRAS tumour mutational analysis of archival tissue was performed for these patients and only those with RAS-WT genotype were included in this report. Figure 1 illustrates the study flow diagram.

Data on chemotherapy and cetuximab components of treatment dose reduction and omission or treatment delay for ≥7 days due to toxicity were captured as surrogates for clinically significant intolerance. In this context, particular attention was paid to capture the grades of cetuximab specific toxicity, namely, skin rash, pruritus, fatigue and diarrhoea. Adverse events (AEs) severity was graded according to the National Cancer Institute Common Terminology Criteria for Adverse Events (NCI CTCAE) V.4.03. Additionally, the rationale for cetuximab dose reduction/omission/delay was captured and categorised into five reasons (refer to the ‘**Results**’ section for more details).

Efficacy endpoints of interest were radiological best objective response rate (ORR), progression free survival (PFS) and overall survival (OS). Radiological response assessment CT scans were mostly performed every 6–10 weeks as per the routine practice of the treating oncologist unless an earlier assessment was clinically indicated. The ORR was interpreted and documented by the investigators as per Response Evaluation Criteria in Solid Tumors (RECIST) version 1.1 criteria through carefully reading and interpreting the radiology reports. PFS was defined as the time from starting the index treatment (first or second line) to disease progression, death or loss to follow-up. OS was defined as the time from starting the index treatment to the date of death due to any cause, or to the date of censoring at the last time the subject was known to be alive. Statistical Package for the Social Sciences Version 20 (SPSS.20) software was used for statistical analysis including Kaplan–Meier and log-rank tests for survival outcome.

## Results

Sixty-one patients with extended RAS-WT genotype fulfilled the inclusion criteria and are included in this analysis (Figure 1). Median age was 53 (27–75) years. XELOX was administered to 26 (42.6%) and XELIRI to 35 (57.4%) of patients in first line 33 (54.1%) or second line 28 (45.9%) settings. Table 1 depicts detailed patients’ characteristics. The median duration of follow-up for all the cohort was 39 months.

The ORR was 54.5% (XELOX: 68.4% and XELIRI: 35.7%) and 50% (XELOX: 42.9% and XELIRI: 52.4%) in the first- and second-line settings, respectively.

**Figure 2.**
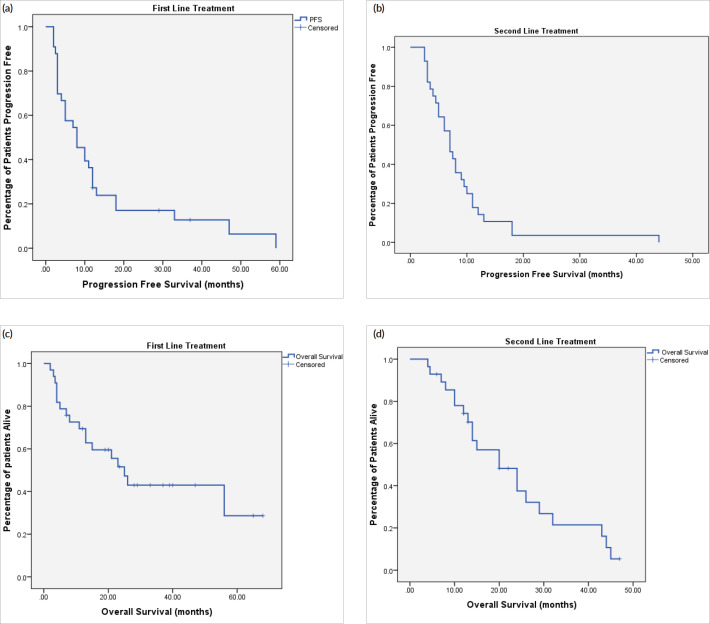
(a): PFS on first-line treatment regardless of the regimen. (b): PFS on second-line treatment regardless of the regimen. (c): OS on first-line treatment regardless of the regimen. (d): OS on second-line treatment regardless of the regimen.

**Figure 3.**
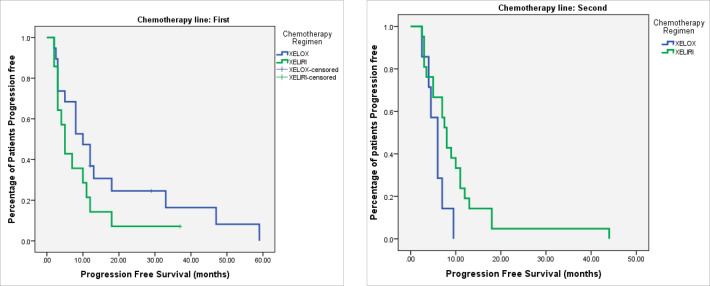
(a): PFS on first-line treatment according to chemotherapy regimen. (b): PFS on second-line treatment according to chemotherapy regimen.

**Figure 4.**
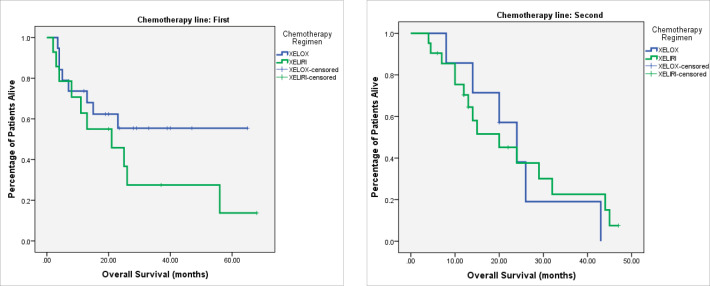
(a): OS on first-line treatment according to chemotherapy regimen. (b): OS on second-line treatment according to chemotherapy regimen.

At the time of last follow-up of each individual patient, 39 patients were dead and 22 were still alive. At the time of final data analysis in December 2020, 9/22 patient were lost to follow-up. The median PFS and OS were 8 and 25 months, respectively, for patients treated in the first-line setting and were 7 and 20 months, respectively, for patients treated in the second-line setting. Table 2 and Figures 2–4 illustrate the efficacy outcome results according to the line of treatment and the chemotherapy regimen.

Chemotherapy component dose reduction was observed in 18 (29.5%) and delay for ≥7 days in 25 (41%) patients. Cetuximab dose was reduced in only 3 (5%) patients, all due to skin rash. Frequencies of chemotherapy and cetuximab dose reduction, delay and discontinuation are depicted in Table 3.

There were no documented grade 4 cetuximab specific AEs. Grade 1&2 infusion reaction, skin rash, pruritus, diarrhoea and fatigue were reported in 0 (0%), 32 (52.5%), 27 (44.3%), 22 (36%) and 27 (44.3%) patients, respectively. The corresponding grade 3 AEs were reported in 1 (1.6%), 6 (9.8%), 0 (0%), 7 (11.5%) and 3 (4.9%), respectively (Table 4).

The median number of administered cycles of chemotherapy was 9 (1–60) and of cetuximab was 10 (1–70). Sixty-one percent and 65.9% of patients received ≥10 cycles of chemotherapy and cetuximab, respectively.

Cetuximab was discontinued permanently in one patient, due to an allergic infusion reaction. Additional 31 patients (50.8%) required dose delay for the following reasons: (a) In eight patients due to toxicities likely related to cetuximab such as rash (4), paronychia (2), fatigue (1) and diarrhoea (1). (b) In eight patients in conjunction with chemotherapy component dose delay due to toxicities related to the chemotherapy such as neutropenia (4), febrile neutropenia (2), thrombocytopenia (1) and hand-foot syndrome (1). (c) In seven patients due to toxicities of undetermined reason ‘could be chemotherapy or cetuximab’ such as diarrhoea (6) and conjunctivitis (1). (d) In five patients due to medical conditions that are unlikely to be related to any of the regimen components such as appendicitis (1), cholangitis (1), intestinal obstruction (1), chest infection (1) and vaginal infection (1). (e) Due to non-medical reasons in three patients who just wanted a short break from treatment.

## Discussion

The substitution of infusional 5FU by oral capecitabine in oxaliplatin and irinotecan containing chemotherapy regimens allows patients to be managed more easily in an outpatient setting. In addition, it reduces the frequency of infusion visits and eliminates the need for central venous access and home infusion pumps, thereby offering a convenient treatment option for patients with mCRC. These regimens are usually administered triweekly [14, 15] with capecitabine administered for 14 days and followed by 7 days of break (2:1 ratio). However, biweekly administration with different dose intensities has been described in the literature and adopted in routine clinical practice albeit on a limited scale [16–19]. Combining these biweekly regimens with biweekly (instead of weekly) cetuximab is supported by pharmacokinetic, pharmacodynamic and some clinical data [13, 20, 21].

At our institution and with the above in mind, we established triweekly and biweekly XELOX and XELIRI regimens for patients with mCRC. In the year 2009, we incorporated biweekly cetuximab (500 mg/m^2^) into the biweekly XELOX and XELIRI regimens to achieve matching and convenient treatment schedules for patients with RAS-WT mCRC. The dose of capecitabine (1,000 mg/m^2^ twice daily) was in line with that in other studies and clinical practice. However, in our biweekly regimen, the duration of capecitabine administration was reduced to 9 days to allow 5 days for recovery before the subsequent cycle is administered. Our intention was to maintain a capecitabine treatment duration ratio of 2:1 similar to that in the original 3-weekly regimen. There is no one standard regimen for the doses and schedules of oxaliplatin when combined with capecitabine and doses of 70 mg/m^2^ on days 1&8 and 130 mg/m^2^ on day 1 in 3-weekly regimens have been described [22, 23]. When combined with capecitabine, the doses of irinotecan are even more varied in weekly, biweekly and 3-weekly regimens. Doses between 70 mg and 300 mg have been described depending on the frequency of administration [15, 24–31].

Considering the above variations, we designed the biweekly regimen of capecitabine (1,000 mg/m^2^ on days 1–9) with oxaliplatin at a dose of 85 mg/m^2^ on day 1 (XELOX) or with irinotecan 180 mg/m^2^ on day 1 (XELIRI) combined with biweekly cetuximab (500 mg/m^2^).

### First-line XELOX-cetuximab

Our real-world experience in routine daily clinical practice has demonstrated the treatment efficacy and safety of biweekly cetuximab and capecitabine based combination regimens (XELOX and XELIRI). In the first-line setting, the ORR to XELOX-Cet was 68.4%. Several clinical trials have demonstrated that the ORR to FOLFOX plus weekly cetuximab varies between 57% and 72% in patients with KRAS-WT and extended RAS-WT mCRC [11, 32–35]. Thus, reassuringly, the ORR observed in our population falls within this reported range.

Our patients who received XELOX-Cet have a comparable outcome to those who received biweekly cetuximab in combination with traditional 5FU and oxaliplatin in relatively large phase II trials. The NORDIC-7.5 trial reported ORR, median PFS and median OS of 62%, 8 months and 23 months, respectively, in 152 patients with KRAS-WT mCRC treated with bolus oxaliplatin and bolus 5FU (FLOX-Cet) [36].

The OPTIMIX-ACROSS phase II trial reported ORR, median PFS and median OS of 60.6%, 10.1 months and 20.8 months, respectively, in 99 patient with KRAS-WT mCRC treated with FOLFOX-Cet [37].

A number of small to medium size studies investigated biweekly cetuximab and XELOX (at times referred to as CAPEOX). In a combined analysis of the FLEET and FLEET2 trials, the investigators identified 88 patients with extended RAS/BRAF-WT mCRC. The ORR was 61.5% in patients treated with XELOX-Cet (*n* = 52) and 66.7% in those treated with FOLFOX-Cet (*n* = 36). The authors concluded that CAPEOX-Cet should be considered as a treatment option for these patients [38]. Similarly, 68.4% of our patients achieved objective responses to XELOX-Cet albeit we did not exclude patients with BRAF mutations. Identification and exclusion of these patients would have likely improved the ORR even further. The multicentre two-arm phase II (SAKK) trial, confirmed the value of adding cetuximab to first-line 3-weekly XELOX. ORR, PFS and OS were 14%, 5.8 months and 16.5 months, respectively, in the XELOX arm and were and 41%, 7.2 months and 20.5 months in the XELOX-Cet arm [39].

In the COIN trial, patients were randomly assigned to oxaliplatin and fluoropyrimidine chemotherapy (arm A), the same combination plus cetuximab (arm B) or intermittent chemotherapy (arm C). The choice of fluoropyrimidine therapy (capecitabine or infused fluorouracil) was decided before randomisation. XELOX with or without cetuximab was administered every 3 weeks. ORR increased from 57% (*n* = 209) with chemotherapy alone to 64% (*n* = 232) with addition of cetuximab (*p* = 0·049) in patients with KRAS-WT tumours [40]. The outcome was further analysed according to the administered fluoropyrimidine. The ORRs were 51% and 57% with XELOX-Cet and FOLFOX-Cet, respectively, and the difference was not statistically different (Odds ratio: 1.26 (95% CI: 0.93, 1.70)) [33].

It is clear from the above data that the ORR of 68.4% to first-line XELOX-Cet observed in our patients compares favourably with that reported in large landmark and other smaller trials.

In the first-line setting, the median PFS was 10 months and OS was not reached in patients who received XELOX-Cet. These results compare favourably to those reported in the literature. In the FLEET2 trial, 14 patients with KRAS-WT mCRC received biweekly XELOX-Cet similar to our regimen except the duration of capecitabine treatment was limited to 7 days (instead of 9 days in our protocol). They reported response in 50% of patients while median PFS and OS were 6.5 and 24.3 months, respectively [20]. The FLEET trial randomised patients with previously untreated KRAS/BRAF-WT mCRC to FOLFOX-Cet or XELOX-Cet. The ORRs in the FOLFOX-Cet and XELOX-Cet were 64.9 % and 72% while the corresponding median PFS/OS was 13.1/38.1 and 13.4/47 months [41].

We appropriately excluded patients with additional novel KRAS and NRAS mutations and this could explain the longer PFS in our population than that reported the FLEET2 trial (10 versus 6.5 months). On the other hand, the exclusion of patients with BRAF mutations may explain the apparent longer PFS (13.1 months) observed in the FLEET trail.

### First-line XELIRI-cetuximab

Combining irinotecan and capecitabine with weekly cetuximab (XELIRI-Cet and CAPIRI-Cet) is effective regardless of dosages and schedules. The AIO KRK-0104 phase II trial randomised 185 patients to 3-weekly CAPIRI-Cet or CAPOX-Cet. The ORR was 50% for CAPIRI-Cet in patients with KRAS-WT tumours and the median PFS and OS were of 6.2 and 21.1 months, respectively. The authors concluded that the addition of cetuximab to either CAPIRI or CAPOX is effective and safe in first-line treatment setting [42]. Similar results and conclusions were presented in an abstract form by another group of investigators [43]. In a dose finding phase I study, biweekly XELIRI was administered with irinotecan dose of 180 mg/m^2^ on day 1 and capecitabine was administered for 7 days at five different investigational dose levels. The identified maximum tolerated dose for capecitabine was 3,500 mg/m^2^/day [44].

As discussed above, different dosages and schedules exist with the XELIRI and CAPIRI regimens. We elected for a lower daily capecitabine dose but over a longer duration (9 days instead of 7 days). In our patients treated with XELIRI-Cet, the ORR was 35.7% and the median PFS was 5 months in the first-line setting. This outcome may be perceived as less than optimum. Possible explanations include (a) relatively small number (*n* = 14) of patients in this group, (b) less than optimum performance status of patients treated in routine daily practice, (c) difference in tumour biology which unfortunately cannot be confirmed as the majority of these tumours were not analysed for more modern biological markers such as BRAF, Microsatellite Instability (MSI) and Her2, and (d) differences in third and subsequent lines of therapy which we are unable to confirm as this information was not required when the study was designed. This outcome cannot be explained by tumour sidedness as only 2 (14.3%) of these patients had a right side tumour or by biological resistance to fluoropyrimidines as only 3 (21%) patients were pre-exposed to fluoropyrimidines in the adjuvant or neoadjuvant settings.

### Second-line XELOX-cetuximab and XELIRI-cetuximab

In our study, 28 patients received XELOX-Cet or XELIRI-Cet in the second-line setting. ORR was 50% while the PFS and OS were 7 and 20 months, respectively. This compares favourably to the results of FOLFOX/FOLFIRI combined with weekly cetuximab reported in the literature. In an extension of the ITACa trial, patients were randomised to second-line chemotherapy (FOLFOX or FOLFIRI) with or without cetuximab. ORRs were not reported. In patients with RAS-WT (regardless of BRAF status), the median PFS was 7.0 months and 3.3 months for chemotherapy-Cet and chemotherapy alone, respectively, with an adjusted hazard ratio (HR) of 0.56 (95% confidence interval (95% CI): 0.29–1.09, *p* = 0.088). The corresponding results for OS were 11.1 and 7.8 months, respectively, with an adjusted HR of 1.27 (95% CI: 0.64–2.50, *p* = 0.489) [45]. The phase II second-line FLIER trial reported ORR in 31.7% of patients with median PFS and OS of 7.4 and 18.2 months, respectively, with weekly second-line cetuximab and FOLFIRI [46]. The ORR was 35% while the median PFS and OS were 7.1 and 14.3 months, respectively, in retrospective analysis of 40 patients who received second-line FOLFOX and weekly cetuximab [47]. In a retrospective analysis of ten patients treated with second-line FOLFIRI and two patients with irinotecan combined with biweekly cetuximab, relative risk (RR) was 33.3% and the median PFS was 4.6 months [48].

### Weekly and biweekly cetuximab

The results of a recent health record-derived database study comparing the outcome of weekly with biweekly cetuximab combined with FOLFIRI/FOLFOX/irinotecan or cetuximab monotherapy in first-, second- or third-line therapy for patients with stage IV or recurrent KRAS-WT mCRC were presented at the American Society of Clinical Oncology Gastrointestinal Cancers Symposium (ASCO-GI 2021). OS in performance status-matched weekly versus biweekly cohorts was not significantly different. The median OS was 23.36 and 29.14 months (log-rank *p* = 0.347) in weekly and biweekly cetuximab groups (each group; 121 patients), respectively, in the first-line setting. The corresponding median OS was 12.93 and 15.39 months (log-rank *p* = 0.504) (each group; 159 patients) in the second-line setting. The investigators concluded that weekly and biweekly cetuximab had comparable effectiveness in real-world environment [49]. The median OS (25 months) in our patients treated in the first-line setting is comparable to these results while the OS (20 months) of patients treated in second-line seems to compare favourably.

The interest in biweekly cetuximab became more apparent with the recent publications (year 2020) of the results of a meta-analysis and a pooled analysis of weekly versus biweekly cetuximab. Both analyses confirmed the non-inferiority of the biweekly regimen and support its use instead of weekly cetuximab [50, 51]. On 6 April 2021, the Food and Drug Administration approved the biweekly regimen of cetuximab at a dose of 500 mg/m^2^ for patients with mCRC and squamous cell carcinoma of the head and neck [52].

### Toxicity and tolerance of XELOX-cetuximab and XELIRI-cetuximab

The combinations XELOX-Cet and XELIRI-Cet were generally well tolerated. The identified cetuximab specific AEs of any grade were rash (62%), diarrhoea (48%) and pruritus (44%) (Table 4). The OPUS trial reported any grade AEs were skin and subcutaneous tissue disorders (90%) and GI disorders (78%) in the FOLFOX-Cet group [32]. The CRYSTAL trial reported grades 3/4 diarrhoea in 15.7% of patients in the FOLFIRI-Cet arm. Grade 3 skin reactions (19.7%), acne-like rash (16.2%) and none of them were grade 4 [53]. The FLEET trial reported any grade acneiform rash in 60% and 70% in the biweekly XELOX-Cet and FOLFOX-Cet groups, respectively [41]. The FLEET2 trial reported any grade rash (80%), diarrhoea (12.5%) while infusion reaction occurred in 3 (7.5%) patients (grade 2: 2 & grade 3: 1) in patients receiving biweekly XELOX-Cet [20].

In a meta-analysis of 47 studies, the rate of infusion reactions in patients treated with cetuximab was 6% compared to 1.6% with the human monoclonal antibody anti-EGFR panitumumab [54]. We found only one patient (1.6%) with documented infusion reaction (grade 3). However, under-documentation of mild grades of infusion reactions could explain our observation.

Fatigue is common in patients with cancer and can be due the disease itself, anaemia, systemic treatment, electrolyte disturbances, radiotherapy, emotional burden and excessive travel for medical care. It can be difficult to identify one cause for fatigue as it is usually multifactorial. A systematic review and meta‑analysis of randomised controlled trials found that anti-EGFR antibodies (cetuximab and panitumumab) increased the risk of all grades fatigue (RR: 1.10, 95% CI: 1.05–1.14) and high-grade fatigue (RR: 1.31, 95% CI: 1.19–1.45). For the cetuximab trials, the frequency of high-grade fatigue was 12.3% in patients receiving cetuximab regimens and 9% in patients receiving corresponding regimens without cetuximab (RR: 1.33, 95% CI: 1.20–1.47) [55]. We report fatigue in 30 (49%) of patients (including grade 3: 4.9% and grade 4: none). The CRYSTAL trial reported a similar rate (5.3%) of grade 3/4 fatigue.

There was no grade IV diarrhoea. All grades & grade III diarrhoea were recorded in 53.8% & 19.2% and 42.9% & 5.7% in the XELOX and XELIRI groups, respectively. There is a general perception that severe diarrhoea is common with XELIRI (or CAPIRI). However, clinical trials did not always show consistent results in this context. For example, The AIO KRK-0104 randomised phase II trial reported grades III/IV diarrhoea in 19.3% and 15.7% of patients on 3-weekly CAPIRI-Cet and CAPOX-Cet, respectively [42]. The available evidence (albeit limited) suggests that biweekly XELIRI is associated with even lower rates of diarrhoea. In a single- arm study, 46 patients received irinotecan (175 mg/m^2^ on day 1), capecitabine (1,000 mg/m^2^ twice daily on days 2–8) and bevacizumab every 2 weeks. Grade III/IV diarrhoea was reported in 7% of the patients [56]. Similarly, only 2% of patients reported grade III diarrhoea and none reported grade IV after treatment with capecitabine at 1,000 mg/m^2^ twice on days 1–8, irinotecan at 150 mg/m^2^ on day 1 and bevacizumab in a Japanese second-line phase II study [57]. It is reassuring that severe diarrhoea was infrequent (5.7%) with XELIRI-Cet regimen in our study. This finding supports the available data that biweekly XELIRI regimens are associated with lower rate of severe diarrhoea and therefore are safe in routine clinical practice.

We acknowledge that retrospective analysis of data collected for a purpose other than the specific study intent has methodological limitations. The presence or extent of symptoms may not be completely recorded [58]. Consequently, accurate reporting of frequency and grades of AEs remains one of the limitations of retrospective studies. Therefore, we decided to use chemotherapy and cetuximab dose reduction/omission and treatment delay as surrogate markers for clinically significant intolerance. Cetuximab was omitted in one patient due to grade 3 infusion reaction and was reduced by 20% due to skin rash in only 3 (5%) patients. Although dose delay was observed in 31 (50.8%) patients, it was needed due to cetuximab specific toxicity in only 8 (13.3%) patients indicating that majority of patients can tolerate this dose and schedule of cetuximab.

## Conclusion

In patients with extended RAS-WT mCRC, biweekly cetuximab in combination with biweekly XELOX or XELIRI is tolerable and has a manageable toxicity profile and associated with favourable outcome. The efficacy of these schedules is comparable to other established schedules considering the heterogeneous population treated in routine clinical practice. Our results add to the already accumulating clinical evidence and support the administration of the biweekly cetuximab regimen. Our findings should reassure oncologists to consider combining this regimen with the convenient biweekly oral fluoropyrimidine based chemotherapy combinations (XELOX and XELIRI).

## Ethics approval

The study has been approved by the Research Institutional Review Board (Research Ethics Committee) at King Faisal Specialist Hospital & Research Centre (Jeddah). Approval number is 2018-46.

## Consent to participate

The consent to participate has been waived by the Institutional Review Board (as it has been deemed that consent would be impossible or impracticable to obtain).

## Authors’ contributions

All listed authors made a substantial contribution to the concept or design of the work; or acquisition, analysis or interpretation of data, drafted the article or revised it critically for intellectual content and approved the version to be published. All listed authors have participated sufficiently in the work and take public responsibility for the content of the manuscript.

## Declaration of funding

The study was financially supported by Merck Serono, Middle East FZ-Ltd, Dubai, UAE, an affiliate of Merck KGaA Darmstadt, Germany (Cross Ref Funder ID: 10.13039/100009945).

## Conflicts of interest

JZ declares acting in advisory and speaker roles for Merck Serono, Saudi Arabia. Other authors declared no potential conflicts of interest.

## Figures and Tables

**Table 1.** Patients’ characteristics.

	Number of patients	Percentage %
Gender Male Female	33 28	54.1 45.9
Age Median (range) years	53 (27–75)	
Primary tumour side Right Left	7 54	11.5 88.5
Organ Colon Rectosigmoid Rectum	21 22 18	34.4 36.1 29.5
Line of cetuximab based therapy First line Second line	33 28	54.1 45.9
Chemotherapy regimen XELOX XELIRI	26 35	42.6 57.4
Line of therapy and regimen First-line XELOX First-line XELIRI Second-line XELOX Second-line XELIRI	19 14 7 21	31.1 23 11.5 34.4

**Table 2.** Treatment outcome.

	First line *n* = 33 (%)	Second line *n* = 28 (%)
Best radiological objective response All Partial response Stable disease Progressive disease Objective response to XELOX Objective response to XELIRI	18 (54.5) 6 (18.2) 9 (27.3) 13/19 (68.4) 5/14 (35.7)	14 (50.0) 10 (35.7) 4 (14.3) 3/7 (42.9) 11/21 (52.4)
Median PFS in months (95% CI) All XELOX XELIRI XELOX versus XELIRI log rank *p* value	8 (3.3–12.7) 10 (5–15) 5 (3.2–6.8) 0.146	7 (5.1–8.9) 6 (4.2–7.8) 8 (6.5–9.5) 0.042
Median OS in months (95% CI) All XELOX XELIRI XELOX versus XELIRI log rank *p* value	25 (17.9–32.3) NR 21 (2.8–39) 0.203	20 (10.7–29.2) 24 (15.8–32.2) 20 (8.4–31.6) 0.864

NR: not reached

**Table 3.** Dose reduction, delay for ≥7 days and discontinuation of the chemotherapy and cetuximab components of the regimen.

	Chemotherapy		Cetuximab	
Dose reduction Number of patients (%)	18 (29.5%)		3 (5%)	
Maximum dose reduction	Maximum dose reduction	Number of patients	Maximum dose reduction	Number of patients
	15% 20% 25% 50%	2 8 6 2	20%	3
Dose delay Number of patients (%)	25 (41%)		31 (50.8%)^a^	

a Cetuximab was discontinued permanently in one additional patient due to an allergic infusion reaction

**Table 4.** Grades of documented cetuximab specific AEs.

	All cohort (%) (*n* = 61)	XELOX-Cet (%) (*n* = 26)	XELIRI-Cet (%) (*n* = 35)
Infusion reaction All grades Grade I Grade II Grade III Grade IV	1 (1.6) 0 0 1 (1.6) 0	1 (3.8) 0 0 1 (3.8) 0	0 0 0 0 0
Skin rash All grades Grade I Grade II Grade III Grade IV	38 (62.3) 12 (19.7) 20 (32.8) 6 (9.8) 0	20 (76.9) 4 (15.4) 13 (50) 3 (11.5) 0	18 (51.4) 8 (22.9) 7 (20) 3 (8.6) 0
Pruritus All grades Grade I Grade II Grade III Grade IV	27 (44.3) 15 (24.6) 12 (19.7) 0 0	14 (53.8) 6 (23.1) 8 (30.8) 0 0	13 (37.1) 9 (25.7) 4 (11.4) 0 0
Diarrhoea All grades Grade I Grade II Grade III Grade IV	29 (47.5) 11 (18) 11 (18) 7 (11.5) 0	14 (53.8) 3 (11.5) 6 (23.1) 5 (19.2) 0	15 (42.9) 8 (22.9) 5 (14.3) 2 (5.7) 0
Fatigue All grades Grade I Grade II Grade III Grade IV	30 (49.2) 14 (23) 13 (21.3) 3 (4.9) 0	16 (61.5) 9 (34.6) 7 (26.9) 0 0	14 (40) 5 (14.3) 6 (17.1) 3 (8.6) 0
